# One Health promotion and the politics of dog management in remote, northern Australian communities

**DOI:** 10.1038/s41598-020-69316-0

**Published:** 2020-07-24

**Authors:** Victoria J. Brookes, Michael P. Ward, Melanie Rock, Chris Degeling

**Affiliations:** 1 Australian Centre for Health Engagement, Evidence and Values, School of Health and Society - Faculty of Arts, Social Sciences, and Humanities, University of Wollongong, Wollongong, Australia; 2 School of Animal and Veterinary Sciences, Charles Sturt University, Locked Bag 588, Wagga Wagga, NSW 2650 Australia; 3 Sydney School of Veterinary Science, Faculty of Science, The University of Sydney, Camden, Australia; 4 Department of Community Health Sciences, Cumming School of Medicine, University of Calgary, Calgary, Canada

**Keywords:** Health policy, Health services, Public health

## Abstract

Community perspectives are rarely sought or integrated into dog management policy and practice. Dog management in remote communities in Australia has focused on reducing the number of dogs, which is often implemented by visiting veterinarians, despite widely-held opinions that fly-in-fly-out services provide only temporary solutions. We conducted participatory research in a group of remote communities in northern Australia to explore how dog-related problems arise and are managed, and explain their impacts from a One Health perspective. Over the course of a year, 53 residents from a range of backgrounds contributed through in-depth interviews with key community service providers, and informal semi-structured discussions with community residents. Free-roaming dogs have broader impacts on canine and human health than previously documented. Dog-keeping norms that enable free-roaming can enhance human and dog wellbeing and intra-family connectivity. This can also cause disengagement and conflict with other residents, leading to resentment and occasionally violence towards dogs. Dog-related problems are underpinned by constraints associated with remote-living, governance and differing sociocultural norms. Focusing on dog population reduction detracts from the welfare benefits and sociocultural value of free-roaming dogs and undermines community-determined management that can overcome constraints to support local values and co-promote canine and human wellbeing.

## Introduction

In this study we explore the impacts of free-roaming domestic dogs and dog management strategies in a group of 5 communities in a remote region of northern Australia. Allowing owned, domestic dogs to free-roam is a common practice worldwide. Contrary to western norms, it does not indicate that dogs lack value^1–3^. Free-roaming dogs are abundant in many remote communities in Australia, where they live in close relationships to humans^4–7^. Almost all dogs are owned and fulfil roles as companions, protectors (both physical and spiritual) and as hunters^7–9^. Dogs also have cultural value, which is demonstrated through the prominence of dogs in dreaming stories, and as totems (both domestic and wild dogs), and their integration in the kinship system through ‘skin names’^10,11^. Rather than being stray animals or a surplus canine population, free-roaming dogs are valued as an intrinsic part of every-day life and are of great importance to individuals and to the social and cultural life of their communities^7,8^.

Clearly human-canine co-habitation can be beneficial to human health and wellbeing, but large and uncontrolled dog populations can also cause problems^12^. Globally, public health authorities have long been concerned about the consequences of free-roaming dogs, with zoonotic disease and bite prevention a particular focus. Better dog management and dog population control is at the core of the drive towards global rabies elimination^13,14^, and the promotion of responsible dog ownership is encoded in a broad suite of World Organization for Animal Health (OIE) policies^15^. Canine rabies is currently exotic to Australia such that the key challenges posed by human cohabitation with an unrestricted canine population include the potential for increased incidence of other zoonotic diseases (e.g. toxocariasis, scabies, ancylostomiasis), bites to people (typically children), poor dog welfare, public nuisance (e.g. barking, forming packs, faecal contamination), habitat competition, and wildlife and livestock predation^16–18^.

Against this background, in remote communities in many regions in Australia, Environmental Health Workers (EHWs) are responsible for animal management. Veterinary services—such as those for reproduction and parasite control—are typically offered on a periodic basis, with veterinarians flying or driving into the community for 2–3 days, a few times a year^17^. Free-roaming dogs and unwanted canine behaviours can be an ongoing source of tension in many remote communities, yet there has been little formal study of the balance of these complex dog–human–environmental relationships, or how daily EHW-led dog management strategies and intermittent veterinary interventions have impacts on the lives of residents^11,19,20^. Globally, program evaluations tend to be unilateral, considering humans as a dynamic population in an otherwise stable system^21,22^. Within the Australian context, the role and importance of EHWs in successful dog management appears to be under-appreciated, with policy and scholarly focus on the veterinary management of dog populations in remote communities^5,23^. For veterinary providers at least, the continuation of clinical interventions is considered to be critically important because dog health can deteriorate rapidly following cessation of veterinary visits, and dog numbers can quickly increase without regular contraceptive measures^23^. However, the combined perspectives of community leaders, EHWs, rangers and remote community members on the purpose and value of dog population management and veterinary dog health programs for remote communities are rarely sought.

In response to the gaps in knowledge described above, we partnered with local leaders and service providers from a group of communities in remote northern Australia to develop a research project. Overall, we sought to understand the perspectives of community members on the purpose and value of veterinary dog health programs and complementary strategies for dog population management. As a team, we were critically aware that Westernized ways of viewing and describing the world are intrinsic to racialized discourses that administratively and politically discount the impacts of settler colonial structures on the lives of Indigenous peoples^24,25^. In Australia, Canada and other settler societies, these structures are not things of the past but ongoing and institutionally embedded^26,27^. Of relevance to our approach, we paid particular attention to understanding local dog management practices and human–canine interactions within broader political structures and their historical, cultural, socio-ecological context. Studies in other settings such as Bangalore in India^28^ and in northern Canada^3,29^ highlight how colonial mindsets and public health concerns can limit the focus of intervention strategies to controlling the negative impacts of free-roaming dogs, while ignoring the beneficial effects of the relationships between dogs and people. Drawing on these insights, we aimed to explore how dog-related problems arise and are managed in northern Australia, to explain their impacts from a One Health perspective, and to cooperate with local leaders and service providers to develop mechanisms for improvement.

## Methods

### Study setting

The study region is in northern Australia and comprises a group of five communities that lie within 15 km of each other by road, and approximately 1,000 km by road to the closest urban center (with a population > 10,000 people). Remoteness is exacerbated by climate; during the rainy season (November–April), access is often by air only because roads become impassable. In 2016, 2,796 people lived in the five participating communities, of whom 87% were Aboriginal and Torres Strait Islander peoples^30^. Recent published estimates indicate that in 2017 the total dog population was approximately 813 dogs (95% range 770–868) of which 254–680 could be free-roaming; that is owned and unrestricted^4^.

The local government council incorporates all five communities. Prior to amalgamation in 2008, each community had its own council. Visiting veterinary services were provided on a periodic basis, directly to each community, until 2012. A veterinary service was re-instated in late 2016, and visits have occurred at 3–6 month intervals since then. Rather than moving between the 5 communities, in the new service a central clinic has been set up in one community. Hence residents from other communities must bring their animals to the central clinic in most cases. Funding from the state health department is directed to animal management depending on local council priorities. Expenses to animal owners are either nil (for example, sterilisation and parasite treatment and prevention) or subsidised (for example, vaccination and treatment for individual disorders).

### Study process

The study process was approved by the University of Wollongong Human Research Ethics committee (approval number 2018/309). The study was implemented according to the approved process (including informed verbal consent rather than written consent) and relevant guidelines. Therefore, all participation was voluntary with informed verbal consent, all participants were 18 years old or over, and all were fluent in English.

Two or three members of the research team (VB, CD, MW) spent a week in the study region four times over a period of 12 months. During the first visit, we focused on scope and development of study design, and introductions to key-role holders in the communities, building on research relationships developed over the previous 5 years^31–33^. During the second and third trips we conducted in-depth interviews and informal and opportunistic discussions with residents. The final visit focussed on dissemination of findings to our research partners (local leaders) and interested community members. This process prompted further feedback on the study and opened up further discussions about the impacts of dogs and the purpose and conduct of veterinary service provision in remote communities. We incorporated these new insights, findings and conclusions into our analyses, and further refined our understanding of how dog problems arise, their broader implications and the influence of different dog management strategies.

Participatory research principles underpinned the study process^34,35^. We regularly consulted with members of the Environmental Health Services in the study communities during study development, implementation and dissemination of findings, to contextualise the research, to represent a range of community views, and to ensure relevance to local residents^36^. Members of the research team had been conducting studies on rabies preparedness within the communities since 2012. In addition to formal connections through research, significant time was also spent in informal conversations with passers-by on streets and with other patrons in local cafes and shops that furthered our understanding of social life within each community^37^. Because some team members are practicing veterinarians, opinions on and treatments for sick and injured animals were occasionally requested by residents and provided to the extent that was possible in situ, both to alleviate animal suffering and as an act of reciprocity.

Two types of interviews were conducted; formal semi-structured interviews with holders of key-roles (occupations relevant to the study) in the community, and more informal semi-structured interviews with community residents. Advice from contacts during our first visit for this study, and experience from our previous studies in the region, indicated that occupations relevant to the current study included animal and human health workers, school teachers and childcare workers, councilors, and local business and hospitality managers. Key-role participants were identified by local research partners and through contacts established during previous research, and were selected purposively to reflect and capture the range of perspectives about dogs and their management in communities. All key-role interviews were arranged by prior appointment, conducted in participants’ workplaces, and were audio-recorded (with participants’ permission). Residents for community interviews were selected opportunistically (guided by local members of the research team), and were conducted in coffee shops, house yards, public spaces or workplaces.

Broad interview topics for both participant groups included dog value (their own experiences as well as community perspectives), and the provision and impacts of veterinary services and dog management in the community. The latter topic questions were tailored to participant group and role; for example, Environmental Health Workers (EHWs) were asked about the logistics and constraints of service provision and management, and community members were asked about their experiences of veterinary services and dog management. Interview guides were used for each participant group so that topics were addressed systematically. Guides are included in online Supplementary Material.

Sample size was determined by progress towards thematic saturation and opportunity to test emergent themes in the later stages of the study. Community members who participated in the study were offered a small honorarium to thank them for their contribution. Participants were interviewed either individually or in small groups, and field researchers (VB and CD) made detailed notes during and after interviews.

### Analytic process

The study and analytic processes were conducted concurrently. Researchers discussed field-notes and participants’ responses, and compared and contrasted insights and emerging themes so that interview focus was refined iteratively throughout the course of the study^38,39^.

Audio-recordings were transcribed by a professional transcription service in a naturalistic style. All data were analysed qualitatively according to the tenets of framework analysis^40^. Initially, authors VB and CD repeatedly reviewed transcripts and field notes, then open-coded the transcripts by themes into (1) substantive items (for example, the veterinary service, tourists, hunting), (2) values and attitudes (for example, welfare and health impacts), and (3) emotions (for example, resentment, violence, acceptance). As analysis progressed, VB and CD developed a framework to summarise data, into which relevant excerpts from either key-role or community residents were entered. We used NVivo qualitative data analysis software (QSR International Pty Ltd. Version 11, 2015) to facilitate data organization, open-coding, and thematic analysis. VB and CD consulted with and discussed emerging findings with MW, MR and local research partners periodically, depending on project needs and individual availability. Drawing on our respective knowledge and experiences of the study setting, relevant literatures and policy context, all authors reviewed alternate hypotheses and explanations were tested throughout the study and during the drafting of this paper to reach consensus on interpretation.

## Results

### Interview sample

We conducted sixteen interviews of approximately 30 min’ duration (range 10–60 min) with key-role participants. All were with individual participants except for two interviews, each with 2 and 3 participants. Seven key-role participants had animal health roles, two were local business and hospitality managers, one worked for the council, four worked in human health and two were educators. Nine of the key-role holders were transient workers with Anglo-Celtic backgrounds who had been contracted to fill service roles through the local council, local area corporation, or government agencies; seven were Indigenous, of whom two had moved to the area from the Torres Strait. We conducted 37 community interviews with participants from all communities; most were permanent residents who had grown up and lived most of their lives in the local area. All were First Nations Australians. Although these participants were not selected based on their work roles, three had worked in animal management prior to council amalgamation in 2008. Median group size was 1 participant, with up to 8 participants, and the duration of interviews ranged from 5 to 25 min.

### Dog-related benefits

The interviewed participants emphasised the value of the practical uses of dogs as well as their value as family members (Supplementary Table 1). Co-habiting with dogs provided people with security by preventing other animals and people entering home yards. In addition, hunting dogs helped people acquire inexpensive and nutritious food for family and relatives (for example, feral pigs, cattle and kangaroo). Some key-role participants also noticed that hunting appeared to improve hunters’ sense of self-worth and wellbeing. Other tacit benefits associated with keeping dogs included companionship and providing a sense of responsibility (‘something to care for’). These beneficial interactions usually entailed a sense of reciprocity, as explained by one participant:

‘…they've been our best friend for, man, from the wild dingo time. So, I think it’s [dogs] everybody’s best friend, to be honest…. If you raise them from little pup, as you know, they’ll look after you.’

Participants spoke of dogs who would regularly visit houses occupied by family members—looking for food and company while their owner was at work or school. These dogs were appreciated as individuals with unique preferences whose roaming in pursuit of their own well-being helped to connect members of the family. Notably, while almost everyone with whom we spoke in a household where dogs were kept, and had positive attitudes towards those particular dogs, none of the interview participants ascribed benefits to dogs who did not have some connection to their own family.

### Dog-related problems

In contrast, other people’s dogs were viewed much more critically, especially dogs that entered their yards, acted aggressively or appeared unhealthy (for example, underfed or with skin disease). The list of problems attributed to other people’s dogs included scavenging (in particular, knocking over rubbish bins), stealing food and harassing people for food, aggressive behaviour towards humans or other dogs and horses, barking (especially at night), the risk of zoonoses and poor community hygiene due to dog faeces (Supplementary Table 2). Of this, a key-role holder noted:

‘Dogs that are neglected and not vaccinated carry disease, you know, they’re potentially going to bite a kid. We’ve seen bites here, it’s not a huge amount, but it’s usually dogs attacking other dogs, people will try to break things up and just the classic dog bite…’

Nuisance behaviours associated with participants’ own dogs included: barking; digging holes under fences; and fighting with other dogs that enter their territory, for example. However, these annoyances were considered to be relatively minor, or in some way justifiable, even though the same activities were identified as problems with other people’s dogs.

### Perceived causes of dog-related problems

In discussions during interviews, dog population size and the ways in which people looked after their dogs were seen as being the major drivers of dog problems in their communities. As people spoke of how dog populations could cause problems in their communities, three underlying factors which constrained both the capacity to care for dogs and control of dog population size were commonly described: ‘remote living’, ‘differing norms’ and ‘governance’ (Supplementary Table 3). People did not focus solely on dog population reduction, or articulate an ideal population size. Instead, they described the balance between these community-based factors and community capacity to support dogs so that they do not cause problems.

#### Remote living

Nearly all participants told us that intermittent and infrequent veterinary services reduced opportunity to sterilise dogs, which led to overpopulation. At the same time, retail prices for dog food and parasite control are higher in remote regions so that residents were not always able to afford basic dog-care products. Community members told us that this situation was not confined to animals:

‘It’s like everything here… lack of services… you know…there’s lots [of that] here… Because there’s no services, dogs don’t get de-sexed. They roam the streets and free-rein and there’s pups everywhere’

The impacts of a lack of access to veterinary services and the high costs of dog-care products were compounded by the relatively low household incomes. Some participants overcame local constraints by accessing health care and cheaper dog-care products in the regional centre (1,000 km away) but this was not an option for everybody. These owners did their best for their dogs by using traditional medicines or products available locally, either from the pharmacy or the EHWs.

#### Differing norms

Human-oriented roaming—such as visiting relatives, or following owners to work or children to the beach—was generally not viewed as problematic (‘my son’s dog comes to my yard and I feed her… she’s a nice dog’). This was associated with participants’ own dogs. However, dog-oriented roaming, such as when dogs formed packs for chasing and scavenging, was perceived as being a significant problem. This was generally associated with other people’s dogs, and by implication, their owners (‘people not restraining their dogs in their own yard; that’s the biggest problem’). When answering questions about their perceptions of how dogs are kept in the study setting, temporary residents reflected on their experiences of other places where they had lived, where dogs were restrained; most said that all roaming (human- or dog-orientated) was a key driver of dog-related problems.

Visitors and tourists—who sometimes fed or adopted dogs because they perceived roaming dogs as unowned and unhealthy—were almost uniformly seen by both permanent and temporary residents as being at best uninformed, or more commonly, lacking respect for the local people. As one community member told us:

‘All these tourists’ll [sic] come up here and carry on and kick up a stink, they have no clue what it’s like here, you know? It’s a different world … just come here and experience it… take your photos and go home.’

Both temporary and permanent residents shared a similar level of annoyance with outside interference. During discussions it became clear that access to interventions to control canine reproduction are valued differently—which had implications for veterinary service utilisation. Most key-role participants who were temporary residents saw sterilisation as an important part of dog care, and consequently, sought opportunities to have the procedure performed locally. In contrast, most interview participants who were permanent residents told us they preferred to delay surgical neutering of their female dogs until they had at least one litter. For hunters, delaying or not performing neutering was pragmatic—puppies are needed to replace their own hunting dogs and also offered for sale—but others simply liked having puppies in their home yard.

#### Governance

Housing in the region is council-owned, and local bylaws state that dogs should be restrained in the yards of individual owners. However, a common response to questions about roaming dogs was to point out that yard-fencing was often inadequate so it was impossible to prevent dogs from escaping or gaining access to people’s yards:

‘They know how to dig under fences … some of the bigger dogs can actually jump over and trouble starts …. If they smell anything in the bins then, yeah, they make a mess for your yard with all the rubbish everywhere. And it’s not one dog. It’s a whole lot of dogs.’

Some participants, especially key-role holders, thought that given the abundance of roaming dogs, the dog pound was not used as much as it should be. Other participants said that impounding dogs was futile because the pound facility was on the edge of town and unsupervised such that:

‘…owners would come in at night time and just cut the fence and let the dogs out. So they’ve [the Council] given up putting them in there.’

From discussions with individuals and groups across the 5 communities it was clear that EHWs have constraints which impede their capacity to enforce by-laws; for example, residents did not always feel obliged to comply with requests from EHWs who did not live in their community. Most people with whom we spoke thought that EHWs currently did the best they could, because as one key-role participant explained:

‘It takes a lot of courage for those animal control guys to take someone’s dog - they're brothers, they’re cousins. ... Everyone here is related to everybody, so you're going to be offending somebody at some stage. So it's tough.’

Similarly, other by-law controls on dog population levels were largely ignored. Nearly all participants were aware of the local council’s ‘two-dog rule’ (two companion dogs/house, or 5–6 hunting dogs if they are restrained in cages) but said that it was not enforced. The veterinary service providers and EHWs independently gave us some insights into the practical difficulties of enforcing the limit on the number dogs; sometimes more than one family lived in a house or a family lived between houses and therefore, restriction of dog numbers by house was difficult. Also, restricting a dog to one yard could mean that a dog went hungry if it was fed by relatives at another house or sourced food by scavenging. Participants perceived that dog-related problems had escalated since council amalgamation in 2008, when environmental health services were centralised and EHWs were no longer community-based. This was seen as evidence that by-law enforcement, as well as veterinary services, both of which were lacking post-amalgamation, were important for dog management:

‘Things were much better pre-amalgamation…Dogs were dealt with within community.’

‘A major difference. I think the bylaws - well, council back then was strict on by the bylaws. So it was two dogs per house. It was really two dogs per house. The regular vets, they did de-sex them. But then the hunting ones had to be locked up properly and registered as well. So you might have six dogs.’

In short, community members, including those who worked in animal management, saw value in stricter bylaw enforcement, but there were overriding concerns that making people confine their dogs would create animal welfare issues and create social conflict.

### The impacts of dog problems

During discussions about dog-problems and how people reacted to and sought to manage them, broader impacts became apparent (Supplementary Table 4). Dog problems incited feelings of anger or frustration for key-role holders and community members. However, rather than risking overt conflict with their neighbours, participants’ reactions to dog problems oscillated between weary ‘acceptance’ and passive ‘resentment’. As we elaborate below, managing the dissonance between these positions created social tension and influenced people’s behaviour in ways that impacted on both human and dog well-being.

#### Acceptance

During interviews participants often mentioned that they threw rocks or chased dogs from their properties. Even so, most sought to portray dog-problems as ‘just part of life’ because they were loathe to confront owners or report nuisance dogs to authorities. The major impediment for action, in the words of one key-role participant, was because:

‘…you'd cause big problems. You could end up in a [fight] - people here will fight over animals. So, you just think, is it worth it?’

In general, they did not perceive that dog control and improved health was something that could be easily changed given the causes of dog problems, and accepted this situation as a reality of living in the region. Overall, acceptance appeared to be a passive response, because participants felt powerless to resolve or avoid dog problems, and did not indicate approval of dog problems and their causes.

#### Resentment

Several key-role participants expressed deep resentment about the presence and pervasiveness of dog-problems. They gave extremely candid accounts of how they would deal with nuisance dogs, both in general and if they were personally negatively impacted by dogs. Mass culling, poisoning of individual dogs, and attacking dogs with weapons (rocks, axes, hot water and oil) were all mentioned. Most of these were hypothetical, and were expressions of anger. As well as an eventual overriding concern for animal welfare, these comments were usually tempered by acknowledgement of the need to avoid confrontation in the communities. Hence participants resorted to condemnation of local dog-keeping practices.

Many participants—including temporary residents who disapproved of local dog-keeping norms—resented new transient residents or tourists who took inappropriate actions to solve dog problems without understanding the complexity of the situation. Some participants reacted defensively; others felt obliged to educate newcomers; and some felt shame about the state of free-roaming dogs because they perceived or heard negative judgements by the visitors about Indigenous Australians, the whole community, or both, either directly or via social media.

### Opinions on dog problem solutions

Everyone with whom we spoke wanted to reduce dog problems. Community dog management, policy enforcement, management of dogs by owners and increased veterinary services were all raised as potential solutions (Supplementary Table 5). How these should be delivered and their relative emphasis differed between participant groups and individuals, but overall, a ‘community-based’ rather than a ‘top-down’ approach was emphasised.

#### Local policy, community dog management and ‘the two-dog rule’

Several participants told us that before the 5 local councils were amalgamated in 2008, problem dogs were not a significant issue. EHWs had worked within their own communities where they knew the dogs and their owners, and participants who had been EHWs at this time recalled that council bylaws (for example, impounding dogs that roamed where they were not welcome) were straight-forward to implement. Consequently, many said that EHWs should be community-based. Key-role participants in particular wanted roaming dogs to be registered so that when they were impounded, owners would have to pay a fee to retrieve their dog. For most, the priority should not simply be reducing the number of dogs; improving the quality of dog care was more important.

#### The veterinary service

Most participants were aware of the fly-in veterinary service but many told us that they did not use it. People told us that they found it difficult to access, or that they were not aware of when the veterinary service was available. Rather than setting up in one community and waiting for people to bring their animals, most of the dog-owning permanent residents believed that the service needs to move between the communities. Free-roaming dogs are not lead-trained and are territorial, and although hunters can transport their dogs on the backs of utility vehicles, other community members do not own cars or do not want to put dogs in their cars.

There was also clear resentment over the apparent prioritisation of dogs belonging to temporary residents, and some key-role workers said that local residents were discouraged from using the service if it appeared busy with these people. Also, the opportunities for veterinary providers to increase within-community skills in animal management were limited by time (due to the unpredictable and short duration of the veterinary visits). There was also a lack of clear definition of the boundaries of veterinary contributions to community dog management such that our perception was that the day-to-day role of EHWs in animal management was pushed into the background due to the focus on the time-constrained veterinary visits and veterinary duplication of EHW-provided services, such as parasite control.

#### Individual dog management

Almost all participants thought that more education about appropriate dog care was needed. Most often, calls for education were associated with participants’ complaints about how other people cared for dogs. A need for education was most often brought up when participants reflected on how people looked after their dogs differently ‘up here’, as part of expressing the opinion that the best way to reduce problems was to make remote communities look after their dogs in the same way that people do in more cosmopolitan settings.

## Discussion

Our findings are consistent with previous studies on the direct benefits and disadvantages of keeping dogs in remote communities, both in Australia and worldwide^1,3,7,19,41^. However, by further exploring dogs’ roles and activities, we find that in the remote communities studied, people’s perceptions of the benefits and disadvantages of dogs appear to be conditional on whether a dog belongs to their family. We use the term ‘family’ in this discussion to refer to Aboriginal and Torres Strait Islander peoples’ extended family structure in which households are often large, compositionally complex and relatives live across more than one dwelling^42,43^. We find that this conditional interconnectedness between dogs and people, as well as the influences of place (location, housing quality, norms and governance), results in broader and more complex reactions and impacts on both human and dog well-being. Focusing on the direct benefits and disadvantages of free-roaming dogs by considering humans and dogs separately and in isolation from their connected social structure fails to capture important nuances and dimensions. Consequently, despite the public and policy prominence of veterinary modes of managing dog populations in remote settings, the solutions to dog problems suggested by participants are multifaceted; solving dog problems is not a simple case of ‘increasing the vet work’ to reduce dog population size in remote communities.

Social, family-orientated roaming is part of the day-to-day events that interconnect people and dogs with the rest of their family; dogs in northern Australia are member of families but lead independent lives and contribute as individuals to the social life of the larger community. In contrast, the social behaviour of owned dogs in many Western cosmopolitan settings—who are nevertheless, considered to be part of their human family^44,45^—is owner-regulated^46,47^. This reduces the dogs’ opportunity to conduct their own human or canine social engagements, but also reduces public health risks. In a study in Chennai, India, Srinivasan^48^ notes the way public health views of dogs and dog-related problems are predicated on colonial notions of control and elimination of risk. Yet dogs are social animals and domestic dogs are considered by some to be ‘social generalists’, meaning that they have evolved cross-species social cognition^49^. This enables ‘pack flexibility’, facilitating the dog–human bond and their successful cohabitation with humans. Traditional modes of dog-ownership in remote Indigenous communities might therefore be more in agreement with modern aims of animal welfare, in which opportunity to engage in rewarding behaviours (canine social-networking) is valued^13,50,51^. However, whilst this networking might support dog welfare and be intrinsic to the interspecies dimensions of family life in some communities, our study confirms that free-roaming dogs can cause problems beyond impacts such as zoonotic risks and dog-bite injuries to people or other animals. Dog-related problems can cause anxiety, foster resentment, and reduce social cohesion which affects the lives and experiences of dog owners and non-owners alike^12,47^.

Conflict is in part caused by differing norms and expectations surrounding dog management, especially between the Westernised expectations of temporary residents and the local norms of dog-keeping practiced by permanent residents. The paradigmatic differences between cosmopolitan and local styles of dog-keeping have concrete impacts. Covertly, permanent residents develop grudges against specific troublesome dogs, and by implication their owners; whereas temporary residents sometimes also adopt racialized discourses to both explain and condemn local dog-keeping norms and practices. Overtly, the overriding response was to avoid interpersonal and intercommunity confrontation. Instead, people directed efforts to ameliorating or avoiding unwanted dog behaviours by reinforcing their defences (improving their own fencing, acquiring bigger dogs), altering their activities (finding alternative routes to avoid aggressive dogs) or becoming themselves aggressive towards roaming dogs. Therefore, whilst family-orientated roaming could reinforce positive social relationships and networks, thus building and enhancing community cooperation for mutual benefit^52,53^, when these same canine behaviours were resented, communication and cooperation deteriorated. In this way, dog-oriented roaming undermined social relationships within the broader community and eroded other norms such as reciprocity and trust.

The intense dog-focussed reactions that we found in the current study highlight the lack of agency that people have in dealing with the drivers and impacts of these problems in these study communities. It is also potentially a manifestation of insecurities and anxieties when the possessive norms of white settler colonialism seem to be ignored or are not easily enacted^54^. Against this background, the OIE^55^ acknowledges that “dog ecology is linked to human activities,” such that “control of dog populations has to be accompanied by changes in human behaviour to be effective.” However, in the absence of a direct and present threat to human or animal health it is arguable that what constitutes the appropriate norms of dog keeping should be negotiated acknowledging local sovereignty in local cultural context. In the current study, we found that whilst policy enforcement married to broader adoption of westernized conceptions of responsible ownership were highly supported by many permanent and temporary residents, top-down approaches focused on imposing strict dog control were not. In our view, a One Health perspective must consider more than the protection of human populations from animal origin disease and injury, by promoting physical, mental, and social well-being for the sake of both animals and people^15,22,56^. In practice, efforts to co-promote health and welfare in humans and other species within a One Health approach almost inevitably involve trade-offs between the preferences and needs of different groups (and species) within a community^15,57^. In remote communities in northern Australia, enforcing Westernized modes of dog keeping that stop dogs from roaming is likely to come at the expense of valued and health-enhancing dimensions within and across extended family structures. Moreover, the imposition of control on dog populations can be construed as an extension of colonial practices and an attempt to regulate Aboriginal life^8^.

There is not a simple solution to dog management under the circumstances described. Nonetheless, we believe that local EHWs have the necessary knowledge and skills to provide a supportive structure; one that enables local dog-keeping norms yet also manages the number and health of free-roaming dogs. Knowing your fellow residents through shared histories and activities is a strength of remote-communities and veterinary contributions to programs need to complement and support local capacities, rather than becoming the sole focus for dog management. This shift requires acknowledging that well-intended interventions framed by Western subjectivities and objectives can disempower local actors and re-entrench colonial power relations. In locations where EHWs or similar personnel exist who can support dog health, veterinarians’ roles should be limited to restricted acts of veterinary medicine. People who work in their own community can provide a less prescriptive and more community-specific approach to reduction of dog problems that supports a healthy dog population within the constraints of remote living, at a size that fulfils individual owners’ needs (for example, social connections and hunting) and enables more traditional dog keeping practices such as roaming, without causing so many problems.

## Conclusion

The lives of free-roaming domestic dogs in remote communities are intertwined with human society, and their impacts, including the way in which people respond to dog-related activities, are dependent on the values, expectations and capacities of people who keep dogs, as well as the place where they live. All owners whom we interviewed felt a responsibility to provide the best care for their dogs, whilst acknowledging that this was difficult or impossible to access given the constraints of remote living. The ripple effects of dog problems (scavenging, chasing and barking) and poor health can therefore cause simmering conflict between residents, leading to resentment and occasionally violence that impacts on human and dog wellbeing. Whilst acknowledging dog problems and the generally poorer health of owned, free-roaming domestic dogs in remote communities, we believe that it is important to acknowledge that dog keeping in remote communities is paradigmatically different from westernized modes of dogs ownership so it is important not to apply a deficit model by imposing rules associated with more cosmopolitan settings. We also suggest that solving dog problems by focusing on veterinary input to decrease the dog population is a single-sided, unsustainable approach to dog management which can undermine and detract within-community dog management. Instead, community-based management should be the foundation from which sociocultural norms can be supported whilst minimising dog problems.

## Supplementary information

Supplementary Information.
