# Supplementary material for: One Health promotion and the politics of dog management in remote, northern Australian communities
Source: Sci Rep. 2020 Jul 24;10:12451. doi: 10.1038/s41598-020-69316-0 (PMC7381604; doi:10.1038/s41598-020-69316-0)
Supplement: Supplementary file 1 — Supplementary Information. [file 41598_2020_69316_MOESM1_ESM.docx]

**Supplementary Material**

**Dog politics in remote communities in northern Australia: from overpopulation to One Health promotion**

Victoria. J. Brookes^ab*^, Michael P. Ward^c^, Melanie Rock^d^, Chris Degeling^a^

^a^Australian Centre for Health Engagement, Evidence & Values, School of Health and Society - Faculty of Social Sciences, University of Wollongong, Australia

^b^School of Animal and Veterinary Sciences, Charles Sturt University, Australia

^c^Sydney School of Veterinary Science, Faculty of Science, The University of Sydney, Australia

^d^Department of Community Health Sciences, Cumming School of Medicine, University of Calgary, Canada

**Interview Guide for Occupants of Key Roles**

**Interview Guide (may cover all or some of these)**

1. What is your day-to-day experience of dogs in the community?

[PROBES: Their perspective on dog numbers and whether dogs are a problem, ask any necessary clarifying questions]

1. What do people who live here do to look after their dogs and keep them healthy?

[PROBES: Their perspective on dog ownership, dog health and whether dog welfare is a problem, ask any necessary clarifying questions]

1. What do most people think about dogs and how they live?

[PROBE: CAN YOU EXPLAIN, TELL ME MORE, PROBE THEIR PERCEPTION OF DOG POPULATIONS, BOTH THEIR OWN INSIGHT AND THE INSIGHT OF OTHER KEY ACTORS, AND HOW THIS MAY OR MAY NOT HAVE COME FROM THEIR DIFFERENT ROLES AND RESPONSIBILITIES]

1. What do most people think about the Vet coming a providing free veterinary services?
2. What role do you play / responsibilities do you have in the community?
3. From your perspective, are dogs a significant issue around here?

[PROBE FOR REASONS: E.G. TELL ME ABOUT THAT, WHAT LED YOU TO THAT CONCLUSION…]

1. From your experiences, what are the key lessons to be drawn on how we should seek to manage dog populations?

[PROBE FOR REASONS: E.G. TELL ME ABOUT THAT, WHAT LEADS YOU TO THAT CONCLUSION…]

1. Thinking prospectively, do you foresee any problems with how dogs are now managed in your community?

[PROBE FOR REASONS: E.G. TELL ME ABOUT THAT, WHAT LEADS YOU TO THAT CONCLUSION…]

1. Is there anything else pertinent to dog populations or how we control / look after dogs that you wish to tell me about?

AT END OF INTERVIEW - *Thank you once again for your time and please feel free to contact using the details on the Participant information statement for an update on the research or should you have any questions*

**Interview Guide for Members of the Public**

**Interview Guide (may cover all or some of these)**

1. What is your day-to-day experience of dogs in the community?

[PROBES: Their perspective on dog numbers and whether dogs are a problem, ask any necessary clarifying questions]

1. What do people who live around here do to look after their dogs and keep them healthy?

[PROBES: Their perspective on dog ownership, dog health and whether dog welfare is a problem, ask any necessary clarifying questions]

1. What do most people think about dogs and how they live in the community?

[PROBE: CAN YOU EXPLAIN, TELL ME MORE, PROBE THEIR PERCEPTION OF DOG POPULATIONS, BOTH THEIR OWN INSIGHT AND THE INSIGHT OF OTHER KEY ACTORS, AND HOW THIS MAY OR MAY NOT HAVE COME FROM THEIR DIFFERENT ROLES AND RESPONSIBILITIES]

1. What do most people think about Vets coming and providing free veterinary services?
2. Are dogs a significant issue?

[PROBE FOR REASONS: E.G. TELL ME ABOUT THAT, WHAT LED YOU TO THAT CONCLUSION…]

1. How should we manage dog populations?

[PROBE FOR REASONS: E.G. TELL ME ABOUT THAT, WHAT LEADS YOU TO THAT CONCLUSION…]

1. Is there anything else you wish to tell me about?

AT END OF INTERVIEW - *Thank you once again for your time and please feel free to contact using the details on the Participant information statement for an update on the research or should you have any questions*

**Table 1: Dog benefits**

Comments relating to cultural significance of domestic dogs were few and have not been included in these tables.

|  | **Community** | **Key-role** |
| --- | --- | --- |
| **Theme** |  |  |
| **Security** | ‘Yeah, they got dogs in their yard that keeping the yard safe, you know from, mmm … break-in… and boys and people that come into your yard without asking.’  ‘But in general, a dog’s good for us up here, for me, is that they’re in our yard, we close the fence at night, it also protects the yard from anything, danger or strangers, or other animals. But especially people as well, so people might want to come in and take some stuff, and also [stealing] as well, stealing and stuff like that. So if you have dogs always a low number of people coming into your yard and taking stuff.’  ‘Watch over [us] at night’ | ‘They'll protect you from snakes and … from other people. They're territorial as well. So, I've got two at home there and no one comes through the gate…’  ‘Have you got any dogs that bark? They said well, you won't get broken into usually, so a lot of people, teachers and people like that will get dogs if they're going to stay a year or two.‘  ‘Well, everybody here has a dog for security. That's the sort of purpose I've got a dog.’ |
| **Hunting** | [Participants with hunting dogs simply stated that they used their dogs for hunting] | ‘Also, they use them for hunting. A lot of people do, as a sport…but, look, I also think for mental health for some of the fellows. It's they take their dog hunting and that's good for them, as well. So, just getting out and about and if that's their sport.’  ‘they use them for hunting, which is good, because they’re used to hunt for pigs… which you know… our pig population is another whole problem, but, if they’ve got their hunting dogs and they’re going out, it’s you know, taking out 3 or 4 pigs here and there, but it’s also providing food for their families… you know… I was surprised at how much meat they can get out of one pig. Cos when you actually see them once they’ve caught them, they are actually quite ginormous, so that’s where if you see some yards with 7 or 8 dogs, you know they are hunting dogs and they are really well looked after because you’ve got to look after them so they go out into the bush and get the pigs.’  ‘They're more useful as a pigging dog because they provide food for the family’  ‘having a series of dogs that can look after you and take you out pigging on the weekend is good.’  **‘**The hunting dogs I see are often very well behaved, very well trained. I've had some down at the gym that are left to sit on the back of a ute. I walked past one day and spoke to them. They didn't move. They wagged their tail, wanted a pat, but they would not move off that ute because they weren't allowed. So hunting dogs are very much looked after, and I guess it's because that is - they're taken out to hunt for bore and pigs and things and so it's a way of providing food for the families’ |
| **Companionship** | ‘I love dogs’ nature. They’re really kind and if you discipline them, they still love you. Like chase them away, they still come back. We all loved dog right from young’  ‘I have pups. Growing them to be hunting dogs. I’m not sure that I want them to be [hunting dogs] they are so cute.’  ‘I think the good points are like that some dogs are friendly and that people own them, look after them, they keep them as their pet’  ‘We love them. We can’t say anything bad about them’ | ‘they do provide companionship to a lot of our community. So, a lot of people are -particularly, I've got some elders that have puppies and things like that. It does give them something to do in a way and something to care for. They've obviously all had children, children have grown up. Now, their children are looking after them but they also just need a bit of companionship. So, I think that's really good.’  ‘Plus there's all the psychological benefits that dogs bring from that. I've got two dogs here myself as well and they're like my best mates, so we run, they get me out of the house, keep me out here.’  ‘they've been our best friend for, man, from the wild dingo time. So I think it's everybody's best friend, to be honest…. If you raise them from little pup, as you know they'll look after you.’  ‘I'm a big believer that dogs add to families. … It's been proven time and time again that to just sit down and stroke an animal is a very calming thing.’  ‘just very good with children.’ |

**Table 2: Dog problems**

|  | **Community** | **Key-role** |
| --- | --- | --- |
| **Theme** |  |  |
| **Aggression. Barking and chasing** | ‘Some of the pig-hunting dogs, they have lots of them. There are lots of them, and if you get another dog, they’ll probably - if it’s in its territory and they’re hungry, they’ll kill the dog, because there’s so much dogs biting them. A lot of the time the hunters have starved the dogs before they go hunting…. so they get more aggressive. The dog sees them, and they find another dog in the area, they just kill it. It’s just the blood and noise, they get really aggressive.’  ‘There’s this man that lives down the road… and his dogs came into our yard the other night, and we’ve been trying to get rid of these other dogs for a while now… and he ripped into my pup just under his front leg… and there’s a big sore…’  ‘Dogs fight when on heat. That’s a problem’  ‘They’re harassing the horses. It’s only a matter of time before they bale up a kid as far as I’m concerned.’  ‘It’s a real problem. The kids have to jump fences to get to childcare.’  ‘Oh they bark at everything….That is a pain. Yep. So screaming and yelling at them to be quiet. They can bark at something so small, could be a little other dog walking past. Because I have four of them now, so everyone’s barking. We have cats as well.’  ‘And yeah, and then they just roam round and you know, they’re outside and they chase them horses.’ | ‘Well, they're a nuisance. They're breeding and breeding and breeding and they just become a nuisance in the community, a lot of dog bites, with multiple dog bites being presented in to the hospital.  It’s mainly adults. Is there usually a story that goes with that that?... they've been trying to move a dog on’  ‘There's a few bites that come through. I would say one every two or three months or just nip - normally it's a puppy. Someone's been playing with a puppy. Because there's so many dogs in the area, obviously the risk of puppy nips is higher, because of the density of dogs, so yeah. You see one quite frequently. ‘  ‘I personally know of a number of dog attacks within the last six weeks. I don't think any of them have been reported’  ‘There's three dogs hanging off - you know, trying to grab a horse.’  ‘Dogs that are neglected and not vaccinated carry disease, you know, they're potentially going to bite a kid. We've seen bites here, it's not a huge amount, but it's usually dogs attacking other dogs, people will try to break things up and just the classic dog bite, usually people trying to break them up. Those dogs aren't vaccinated, they're not well looked after.’  ‘the dogs start barking at the horses and then all the other dogs start barking, so nobody's getting a good sleep here…. All the dogs here, you don't hear the dogs during the day because they're all sleeping, waiting for tonight.’  ‘The noise is a problem. Training three dogs to shut up when they're told is hard enough. If you've got 15, you've got no hope.’  ‘My biggest issue a while ago, 18 months 2 years ago… was a few mobs of dogs were ripping horses apart.’  ‘That's the worst thing, especially they bark - especially the other stuff with cats, horses and all that. So they bark all night.’ |
| **Hygiene and Disease** | ‘You can get ringworms, scabies from dogs…. Sometimes when they poo and you know kids with no shoes or you know, even the parents, and they walk on the droppings and that might cause bug that gets into your body and you get really sick… rheumatic fever.’  ‘Obviously there’s the ticks problem. There’s a flea problem, and there’s a pooing problem, they poo everywhere.’  ‘They got their diseases, we got we diseases.’  ‘Yes, you can catch things from them cos of my partner had to break up two dogs from fighting and well then, hurt himself and now he’s in a really bad situation’  ‘I seen a couple of people sick from the mange. Very rare. ‘  ‘there’s dog shit everywhere … you get all sides of it… dogs die on the side of the road and they’re there for two or three days before anyone’ll pick’em up.’ | ‘If you look at where we sit in relation to New Guinea and Indonesia where there is rabies, I mean it hasn't really made it to that side yet but it is a potential risk factor because the locals do have contact with [Papua] New Guinea, so it is a bit of a gateway. I guess it potentially could be quite a [risk] - introducing diseases and things like that in here.’  ‘I actually asked the doctor about scabies because we all thought you got scabies off dogs and apparently you don’t you get ‘em off kids and we’re not sure how the kids get ‘em, but somehow the kids get ‘em then they spread ‘em to their little friends and if we’re not careful with our hygiene then we can catch it. So we’re all blaming the dogs and it’s not the poor old dogs fault, it comes from somewhere else… I’m not sure where, to me, I’m not sure how it works whether it comes out the ground’  ‘There's some recent research about school sores and other things that children get. They're apparently related to the - they've now decided that's related to the same mite that causes mange in dogs. There's hydatid tapeworm, because these guys are running the countryside eating offal. If they're not regularly wormed and - systemic hydatid cysts, that'll kill you pretty quickly.’  ‘we've got endemic problems with mange, fleas, internal parasites’  ‘Our kids here, the older kids are more aware of they see an unhealthy dog or a dog with sores or anything they're not going touch them.‘  ‘But it’s not fit for little children to be running around in dog shit. I don't know what diseases or whatever the children can have but it's a risk, pure and simple.’ |
| **Scavenging** | ‘As long as my fence is secured I’m alright. I don’t like them coming into the yard scavenging everything, turning bins over …’  ‘Sometimes they tip over bins. A big problem up here, they go after the school and grab them lunch bags from the school.’  ‘you know... ratting in bins… and making a mess, you know’  ‘They knock the bin down and get into the bins, they dig the ground, make holes everywhere. They eat everything. So if you’re turning around they just eat it. They find a piece of rubbish and bring it into the yard, they’ll eat it, and make more mess. They’ll bring meat into the yard too, the other families are cutting up [unclear], they’ll take the chunk of meat into the yard, they eat all the meat.’ | ‘They're hungry, they're looking for the rubbish bin. Then the dogs follow them and bark. Starts off with one dog and then all of a sudden you've got about 20, 30 dogs following the horses.’  ‘They'll go and purchase, or grab dogs, take them home. Then after a while they can't care for them, eh, they don't feed them and all of that stuff. So, that dog, leave their yard and they'll go and knock the bins over. Go into someone else's yard and go and cause trouble, but it's actually not the dog's fault.’  ‘the kids are leaving their lunch outside and the rats [dogs] come out and their bags are gone’  ‘There's heaps of bin knockers around, especially with the horses helping them as well. But once there's a free bin standing there and they can smell food they will knock it down…. That's when the plastic bags and all the rubbish flies around’  ‘They know how to dig under fences, so I've had to put sheets of iron cut to length. Then you seal all the bottoms, because they get in. Some of the bigger dogs can actually jump over and trouble starts, because you can't leave food outside. They'll eat it, or if not the biggest ones, they knock bins down and just total make a mess. A lot of the fencing here are inadequate so the dogs just walk straight in basically. If they smell anything in the bins then, yeah, they make a mess for your yard with all the rubbish everywhere. And it's not one dog. It’s a whole lot of dogs.’ |

**Table 3: Causes of dog-problems**

Mainly from KRs; they are aware of differences.

|  | **Community** | **Key-role** |
| --- | --- | --- |
| **Theme** |  |  |
| **Remote living** | ‘[Dog food is] very expensive…a big dog biscuits and 4 tins, that’s probably like a hundred and something dollars a fortnight rough… yeah, money isn’t good up here anyway… it’s too dear.’  ‘[I buy] the cheapest biscuit.’  ‘My old fella; he uses bush medicine… he has to look which one’s good and go searching the area’  ‘It’s like everything here… lack of services… you know…there’s lots [of that] here…’  ‘Because there’s no services, dogs don’t get de-sexed. They roam the streets and free-rein and there’s pups everywhere ‘  ‘They don’t treat them. They just let them go astray. Yeah.’  ‘I take my dogs to [***] for vet things… ‘  On a cat attacked by a dog: ‘So I grabbed him, took him to the house, he just laid there. I think he pierced his lung I think; he lay there for three days, just laid, and he was breathing really heavily. We prayed and prayed, and three days he came all right.’  ‘think if we had the vet here more often, I think more dogs would end up getting de-sexed’  ‘Oh man, it’s a big issue. It’s our biggest issue in community right now, is not having a full-time vet. I know it costs a lot of money, but vet down south costs a lot of money. But man, it would be so good if we had a vet up here full time. We’ve got so many animals up here, beside dogs.’ | ‘Others probably haven't got enough money for to feed the dogs and themselves. I don't know whether the dogs when they take them pig hunter, whether they keep the pigs to feed the dogs. I don't know. There's people who do look after their animals real good. There's others who can't afford it and just let them roam…. I mean, really when you see the dogs that are not looked after, they're not looked after, eating what they can eat and there's a lot of them in the dump down there. In the scrub. There's a lot of dogs in there, so when the garbage truck comes in there, they're all – at night time – they're all down there. Yeah, it's hard.’  ‘I believe there's been ownership there before and it's become too hard. Whether it's attending to a medical issue or tending to something or it could be a combination that's okay there's one less food that we need to buy for the family. Maybe that's the dog.’  ‘Probably something that needs to be improved is around the food for the dogs as well.’  ‘Sometimes when I catch pig, [if] it's not that fat, feed it to the dogs.’    ‘Without a vet down there, it's impossible, eh….De-sexing is, I think, will be the most [little] way to actually keep the population down and educating the owners as well. Yeah.’  ‘There used to be a vet on [***] but it's pretty expensive to go over there. You have to pay for the ferry which is $146 dollars return. Then vet fees.’  ‘I started encouraging people to get down there [the ‘Environmental Health office]]… because a lot of people look at the cost of the worming tablets [in the pharmacy] and they're really expensive.’  ‘It is just part of being here. A lot of these happen after-hours and a lot of them are pig dogs’  ‘But with myself, yeah, every time I go to [Regional Centre], I get worm tablet, heart tablet and every other stuff. So, I treat my own dogs.’  ‘Well, firstly, [de-sexing is] too expensive. Secondly, I haven't seen a vet here for the past four, five years...’ |
| **Governance** | ‘People not restraining their dogs in their own yard. That’s the biggest problem.’  ‘Council should get together and restrict number of dogs.’  ‘When the 2 dogs rule came in, they enforced that. There was hardly any dogs around. No stray dogs.’  ‘[There’s a] lack of control and care… mainly… no care … and no veterinary service… which is massive.’  ‘It was good in the 80s. They didn’t have dogs jumping in bins then, they were much better controlled. These days, owners let dogs breed and there are pups everywhere. Could only have 2 dogs then.’  ‘Things were much better pre-amalgamation…Dogs were dealt with within community.’  ‘for hunting fellas like, got to write letters… then can keep for hunting, like 4 dogs, but build something behind [the house] for them dogs, make little a platform.’  ‘Back in the [individual community] council day it was all right. Not now I think it’s a bit hard’  ‘[We need] shelter for the dogs, you know? Pound for dogs, you know, stray dogs they can go in there, they get looked after.’  ‘These days, owners let dogs breed and there are pups everywhere. Could only have 2 dogs then.’ | ‘I think, like I said, the amalgamation didn't help us at all. We're very proud communities and everybody looks after one another. If there's a stray dog it will either be put down or taken back to his owner and all that. So trying to get that back into community is the hardest thing.’  ‘But I know I've talked to the environmental health, they'll tell you in the beginning they locked up the dogs in the compound - wandering - in the dog pound, and the owners would come in at night time and just cut the fence and let the dogs out. So they've given up putting them in there.’  ‘People would have multiple dogs, it's crazy. So no one owns them and half the time no one actually legit owns those dogs, they just hang around. [Community X] has big packs, like you see them roaming around everywhere just being dogs, they form these packs and they'll just run in the bush and they must be eating something somewhere. You know, foals, when a foal is born they see them trying to get the foal.’  ‘We've got a fencing program that we have putting fences around all the houses now. But dogs dig holes, don't they, and they learn how to jump.’  ‘A lot of the fencing here are inadequate so the dogs just walk straight in basically. If they smell anything in the bins then, yeah, they make a mess for your yard with all the rubbish everywhere.’  ‘But I think - we don't have a pound. I think if we got a pound now, we would try and get these dogs into a pound and do the old, is this your dog, come and grab it or we'll put him down.’  ‘Well, council's going to put policy of two dogs per yard. That's not happening. Not being policed, I should say… but I know I've talked to the environmental health, they'll tell you in the beginning they locked up the dogs in the compound - wandering - in the dog pound, and the owners would come in at night time and just cut the fence and let the dogs out. So they've given up putting them in there. Then the same dog just wanders around again.’  ‘It was really two dogs per house. The regular vets, they did de-sex them. But then the hunting ones had to be locked up properly and registered as well.’  ‘Yeah, yeah. You've got to. It's ridiculous, somebody got a truckload of dogs. What for? If you want that number of dogs then you need to keep them secure 24 hours unless you take them out somewhere. But yeah, they should enforce the law, because they're not only a nuisance knocking over bins and stuff. But, and I said earlier, they are a danger to other dogs and children, adults too.’  ‘The more people that actively get involved in relations to management of dogs, numbers of dogs, whatever, then the minority will have no other choice but to abide by the ruling.’  ‘and they're a little bit lenient, like if you put them in the pound, if people come and get them, maybe [give them] a fine at first’  ‘But the problem is the overcrowding in the houses as well and you might have sixteen or seventeen people living under one roof and they've all got a dog. They all own a dog, so they're entitled to have a dog. Also you can't really say, two dogs per house, when a whole family live in the same street and dogs go between all of those households. You can't register dogs per house. Oh, that's - because they all kind of get fed by grandma, or auntie. If Auntie doesn't feed the dog and it stays at that one house on a chain, reality is it might not get food and water.’ |
| **Differing norms**  Mainly from KRs because they are the ones who are aware of the differences. | ‘My grandfather always killed puppies when he was - when they were born. Kill them straightaway. You couldn’t maintain them… I was like, why granddad? Then I realised - like I'm older, because they can't maintain them all.’  ‘All these tourists’ll come up here and carry on and kick up a stink, they have no clue what it’s like here, you know? It’s a different world’  ‘They don’t treat them. They just let them go astray.’  ‘People, they get dogs, but they don’t love them enough to keep them in their yard, or they just let them wander off and they become stray and … I think they like them when they get pups and when they get older they don’t care about them..’ | ‘Dogs are around. They're allowed to roam. They - that's the way animals have been for years and years and years.’  ‘It's just the way it is. The packs of dogs roaming around has been just the way it is. What is starting to change is that …the numbers are increasing as well.’  ‘We haven't got a long-term culture here where you worm your dog, flea your dog, whatever, at regular intervals.’  ‘Also there's that normalised appearance of dogs in these communities. When they start looking better, the locals notice it and they comment on their dogs looking better.’  ‘When the tourists come along, they go oh, poor dog. Poor dog. We'll just dump a whole bag of food for the dog…and it just attracts them’  ‘She [tourist] went and took them to the airport. The owner turned up and abused the hell out of her for taking her dogs away.’  ‘The number of dogs tipping bins over and wandering and the tourists do see it. They see old dogs with mange and we've had tourists jumping up and down and ringing [RSPCA]. Then they say, we cover like, it's too far away and its, yeah. You've got environmental people are there to deal with that. Because what they see is, they see dogs with mange and skinny as anything’  ‘There's a lady there who actually rang up *Channel Nine* to come up. There was a dog - I think a dog got lost when he went pig hunting, came back and crawled under one of those villas. She spotted it, and probably - of course she feeds them so the dog hangs around. It's scrawny and everything and she's come up to me and demanded that we do something.’  ‘Tourists and dogs are a big deal. Tourists trying to do the right thing and causing disasters with dogs is a problem, like when they see all the stray dogs outside the Ibis supermarket and decide to be really good and buy a bag of dog food and tip it out in the car park. The dogs all proceed to tear each other limb from limb over the food, so then you've got a bigger problem.’  ‘If you said to most people up here, oh, hunting's bad and here's a PETA t-shirt……they'd look at you and go, what do you mean, people shouldn't hunt? What planet do you come from? It's just what you do.’  ‘Tourists come up and you hear them on the radio, on the UHF and they're like, look at these dogs…’  ‘If I was living in Sydney, would my dog just be allowed to wander around without being on a leash? Is he allowed to just go wherever he wants?’  ‘Why is it different than I if I go into Townsville or Cairns, why can't my dogs roam around? What's different to here? What makes this place so special?’  ‘If I had a dog, there's absolutely no way I would ever take it out pigging. I find it sad seeing these animals ripped up, in an environment where you can't stitch them up and they just get shot, I think it's awful. I don't like hunting, but that's my opinion. I can understand why different people would have a different opinion to that. Under that circumstance, having a series of dogs that can look after you and take you out pigging on the weekend is good.’  ‘I know within the pig hunting community, from what I've seen the dogs are expendable. People don't get too attached to them because it is a dog-eat-dog kind of sport - pardon the pun - and they are kind of expendable. So look, I don't see them getting the best of care.’  ‘it's one thing that I notice every time I'm home I just look at the dogs and go, wow, that's what a dog is supposed to look like. Then I get back here and I'm like, wow and this is what we have here.’  ‘It's normal that there is a bunch of dogs that just hang about.’  ‘I haven't heard that. I - when I start talking about it with people I get this funny look. It's almost like a, but we want it to have puppies, because they're puppies, almost. It's - everybody loves puppies’  ‘Often they'll go to a party on the weekend and the whole family will come there for a puppy each.’  ‘animal control and de-sexing and things like that isn't a big thing. So we have litters of puppies all over the place.’  ‘But a lot of people buy dogs from down south and they buy particular breeds for hunting purposes. Trouble is, they don't look after them and that’s the thing.’  ‘People tend to - they'll go and purchase, or grab dogs, take them home. Then after a while they can't care for them, eh, they don't feed them and all of that stuff. So, that dog, leave their yard and they'll go and knock the bins over. Go into someone else's yard and go and cause trouble, but it's actually not the dog's fault. It's actually the - what'd you call it - the owner's fault, eh, for not looking after and feeding it.’  ‘You've got - the dogs are roaming, so we need to look at putting some sort of cap in there somewhere that - how many animals can you keep on a property of a certain size. If you put food in one end, stuff comes out the other end, and they're all contained in a single residential block. There's problems with that. The noise is a problem. Training three dogs to shut up when they're told is hard enough. If you've got 15, you've got no hope.’  ‘Yeah, look, it's very difficult. I know, to be honest, it's one thing we struggle with all the time. To take those, what we might consider the norms of local law, behaviour of a small town in Australia, to even impose them in a minor way …. just doesn't see to work’  ‘I noticed that every time I'm home, there's such a difference between the behaviour of the dogs here, dogs you don't know, and the dogs of other parts of Australia. That's a bad thing. These things are scared.’ |

**Table 4: Impacts of living with dog-problems**

|  | **Community** | **Key-role** |
| --- | --- | --- |
| **Theme** |  |  |
| **Acceptance** | ‘There probably could be more control here. That’s gonna take time, it’s not gonna happen overnight.’  ‘The horse is just ripped to bits… you know… but that’s me… I found that upsetting … I guess, I dunno, I don’t want to judge… it’s just the way it is… grin and bear it.’  ‘We had a mindset that you were just out of that community and was a bit hard for another person to come and give advice to you, you were not accepted because they had the boundary, but today it’s open now.’  ‘Lots of dog bites going on here… it’s not really anyone’s fault… it’s just the way it is here…nobody’s blaming anybody…’  On dealing with neighbour conflict over dogs: ‘Just close up [adamant]. Ignore it, waa [yes].’  ‘I don’t think change is happening real fast, but I don’t think you can expect things to happen too quick up here…’ | ‘It's just one of those things about living here, it's all very diplomatic; you have to tread with eggshells sometimes. For my safety and other people's safety too.’  ‘Well, they're already attacking people, why aren't you reporting it? We do, but nothing happens. They don't formalise a report, they'll tell the guys when they're driving passed, that dog is biting people, and the guy is like, oh.’  ‘There is the programs that are run, but people just don't partake in them or think they don't need to. I don’t know whether it’s education or it's just the way they are.’  On whether people just live with dog-problems: ‘Yeah. But nobody seems to be doing anything about it’ |
| **Resentment** | On cross-community dog control: ‘Who are you? You’re from another place!’  ‘They’ll grab a dog and take a dog hunting. And if it gets lost they leave it out there and it might probably make its own way home….. And they end up bloody arguing and fighting over them.’  ‘I told them … to get rid of that mangy dog. It’s still roaming around town. You better tell them that if not I’m gonna shoot it and feed it to the crocs.’  ‘Some people don’t take it seriously’  ‘If it’s really a problem, get help or we’ll kill them ourselves! ☺’  [Throwing rocks at nuisance dogs was a very common comment from community participants]  ‘All these tourists’ll come up here and carry on and kick up a stink, they have no clue what it’s like here, you know? It’s a different world… just come here and experience it… take your photos and go home… don’t come up here and jump up and down and say things don’t happen and this is wrong and it’s cruel and all that shit … it’s none of their business, you know?’ | ‘Often you get these kind of sweeping comments and it will come from the communities itself, the mayors, the mayor or the councillors. Well, would you euthanize half our dogs, or - yeah. So, there certainly is tension at times. Of course it's not their own dogs, it's their neighbour's. They're happy to see that [unclear] euthanized, so long as it's not their one. Yeah, look, it is a cause of tension across communities.’  ‘I think if a vet opened up here full time they would be over the moon about it, I think they probably feel a bit ripped off, like most of us live here, it's like, oh we just need more services. We need more of everything… I think they'd feel a bit more loved.’  ‘Well, yeah, I mean, you answer this question; if I was living in Sydney, would my dog just be allowed to wander around without being on a leash? Is he allowed to just go wherever he wants? There's a reason for that, isn't there?’  ‘What makes here so special that you can abuse animals and you can get away with that stuff?‘  ‘Why is it different than I if I go into Townsville or Cairns, why can't my dogs roam around? What's different to here? What makes this place so special? It's not, and there's a reason that we have control for animals, public health, public safety, animal safety. It's just one of the things, you know?’  ‘My neighbour there, you go into his yard and take his dog. That goes everywhere then. You're going to get beat up something bad. They don't give a shit about the number of dogs you should or shouldn't have. If the dog causes harm to someone else or to the other animals, you just keep away. You just put up and shut up, really. Because if you're going to challenge, you're going to get hurt.’  ‘I'll tell you right now, if my cat gets killed by one of these dogs, man I will take an axe to that dog and I won’t have any hesitation in it. If I find out who owns that dog, I'll fucking take him out as well. I'm sick of it.’  ‘These things are scared. They're scared of people. They've had so many rocks thrown at them, they've received so many kicks, they're just not happy.’  ‘I think the wives of the guys just getting sick of the number of dogs in the yard. The biggest comment is - from a lot of the women or the wives or girlfriends of the guys - is they're sick and tired of backyard full of shit. Everywhere, it's ridiculous.’  ‘But then you realise, wow so the only reason why it's not worse than that is that when the litters of puppies are born they’re all drowned. Then 14 months later there's another litter born – it’s drowned. You sort of think to yourself, well, how can I - what sort of people are we?’  ‘When they gang bang your dog and kill it, especially pig dogs, yeah I got upset. I was out for revenge. I wanted to smash fluro - the fluro lights and then mix it in with mince and then give it to them, so they have an agonising death, because I love my dog. But then after, I thought, I even tried chocolate to feed them. They reckon chocolate's not good for dogs. That didn't work. I think that's a lot of bullshit, because they ate that really dark chocolate and then - bastards they're still running around.’  ‘Oh, I could have spoken to the environmental health worker, which lives in [Community X]. But at the end of the day, they work for the council and what could they do? They definitely would not be able to put the dog down, because they're hunting dogs and number two is you'd cause big problems. You could end up in a - people, adults here will fight over animals. So, you just think, is it worth it? No. Is the revenge worth it?’  ‘People are protective of their animals so, therefore, to retaliate or to get revenge really is not worth it because you end up in all sorts of problems. You just get a bigger dog and more dogs and let them fight it out.’  ‘Oil and water, boil it up and you just wait, because they know where the bin is. That does it. Usually they put - tease the dog, put it where the balcony is or somewhere where you can sneak up. They'd be in your bin there and they would - and then they never come back again’  ‘His [dog] will be running down the road chasing a kid on a bike, like trying to grab his leg, and he just sits there. I yell out, tie-up your fucking dog. I'm going to go to the cops and say, let's knock that dog because - and I don't want to do that, I love dogs, but then I have to live across the road from this guy that I've just had his dog destroyed. Everyone in the street would think I'm a hero, but when he gets drunk, it's two o'clock in the morning and I'm coming back ... we all know what's going to happen to me.’  ‘They make a mess for your yard with all the rubbish everywhere … and it's not one dog. It’s a whole lot of dogs. Always, and then the thing I hate, and to tell you the truth, I keep two bottles of Black & Gold Domestos. Even for my - the dog I've got now, because they shit in the yard, everywhere. I'm a person, I love my yard. But when I see dog shit, if it's mine, I don't mind. I'll get the old Domestos, pour it on, let it soak and then flush it into the ground. Other dogs piss me off, because I've got grandchildren that run around in the yard.’  ‘Locals tell me, complain to me about the dogs all the time…. they fully want the animals taken care of. They hate it. They tell me, someone should do something about these dogs. I hate these dogs.’  ‘It takes a lot of courage for those animal control guys to take someone's dog - they're brothers, they're cousins. ... Everyone here is related to everybody, so you're going to be offending somebody at some stage. So it's tough.’  ‘But the overwhelming majority of people that I talk to in community hate the dog problem, they want it fixed, and the horse problem. Not that they hate dogs or horses, a big part of Indigenous culture are both those things, but they just - they hate the neglect and they're [upon] it. Why can't we get this done, because everyone seems to be supportive of it?’  ‘There's this terrible dog down the road, I want to take it off them and ring the RSPCA. Just those attitudes, which I know that they're meaning well, but it doesn't do us any favours in the long term. Because again, they're seen as someone removing an animal from a situation and rescuing it.’  ‘I said, well the only thing we can do with that dog… is knock him over. She starts jumping up and down and carries on. I said, well that's the only alternative. She said, I might take him home. I said, you want to, you can take the other 20 dogs that look that that too if you really want. She ended up ringing *Channel Nine* and RSPCA, reporting all that.’  ‘Tourists and dogs are a big deal. Tourists trying to do the right thing and causing disasters with dogs is a problem, like when they see all the stray dogs outside the Ibis supermarket and decide to be really good and buy a bag of dog food and tip it out in the car park. The dogs all proceed to tear each other limb from limb over the food, so then you've got a bigger problem.’  ‘tourists come up and you hear them on the radio, on the UHF and they're like, look at these dogs…they don't look after - you hear them on channel 40 and I have to go, hey guys, you know everyone here has a UHF, just because you've come off the PDR doesn't put you in a cone of silence … like shut up!’  ‘But it's just like I'm sorry guys, you guys come here, you think you're doing the right thing, you're looking after an animal for three to four months, but then you leave. So, the new person turns up and it's just like, what are all these dogs hanging around? They stink. …They're everywhere, they shit everywhere. …That one's loaded with fleas and ticks.’  ‘Well, if you look at remote communities, it’s how you're perceived. Everybody - the media puts a different - walking stray dogs and what Indigenous communities look like.’  ‘Media, as we know, media puts things out differently to make us look bad. But really until you come to town and see what problems we've got then you can put it out there. So that's what I'm worried for.’ |

**Table 5: Suggested solutions to dog problems**

|  | **Community** | **Key-role** |
| --- | --- | --- |
| **Theme** |  |  |
| **Individuals** | ‘I think there’s more education needed. Especially for the pet owners. As you know education is one of the main things. Change our mindset…’  ‘I think would be good [to have] education around what makes a dog happy, what you would - that they’re not allowed to eat chocolate, stuff like that. No one knows nothing really, they’re just guessing half the time.’  ‘I think education for dog owners, how to maintain dogs. We don’t really know much about dogs, like a professional level. What dogs are like, and know how to groom dogs’ | ‘I think the thing is to educate people here about the dog issue, dog management. Because you do have a lot of people complaining about dogs, but they won't go the next step and put them [down]. You know they [say], oh the environmental department of the council is not doing their job.’  ‘I suppose I see it when I go out, it’s about educating the community, the families about getting in and being proactive before they get their dogs and the purpose behind it and saying that their services are - I didn't know that the staff … at animal control there, what resources they have out there as well. If people knew that they would be a bit more proactive in looking after their dogs.’  ‘The more people that actively get involved in relations to management of dogs, numbers of dogs, whatever, then the minority will have no other choice but to abide by the ruling.’ |
| **Veterinary service** | ‘More often, more advertising’  ‘But I think if we had the vet here more often, I think more dogs would end up getting de-sexed…’  ‘Maybe have a vet clinic based up here that could look after the dogs and things.’  ‘Sometimes when the vets come up they do somebody else’s dog first instead of locals. That’s the thing there. Then the locals start go away from them vet. They put name down council office but when the vet come up here they do somebody else dogs first. They don’t follow the list what they have.’  ‘Our biggest issue in community right now, is not having a full-time vet. I know it costs a lot of money, but vet down south costs a lot of money.’  ‘Nobody can get to them…if they have no car… even in the same community, you still have to have transport… From here, from this street, and I know my dogs have their territory, and take a claim of yards as well, my dogs, so they basically took the next door, that’s their territory, and that’s someone else’s yard. They don’t have dogs, if any dogs on the other side come in they just ambush, chase them away, bite. But last time they had a big fight.’  ‘People don’t want to go to the sewer pond!’ ‘Should be door to door stuff!’  ‘I’m not sure if everybody knows about it. They got posters out on that, on the vet visit.’  ‘They know it’s free but not when it is.’  ‘Some people don’t have transport.’  ‘We don’t know the vet’s here.’  On advantages of having a vet: ‘Yes. To worm and all that. And it’s saving us time walking in the scrub to look for the medicine.’  On communication that the vet is coming: ‘Facebook’s a pretty popular tool here.’  ‘You need everybody to realise and gradually implement their own ways, you know’  ‘They do worming and that, so they give out tablets for worming, ticks, microchips. And that’s probably like every once in a blue moon they come up, but sometimes they can’t make it. They do announce it over Facebook, but yeah, sometimes it’s like a wait… and then we have to book in and it costs… you have to pay…’  ‘They usually put out flyers and posters on shops and things that they are coming … and the council notice board… you know, that we could ring them.’  On cost-free services: ‘Are they?’ | ‘Up until the last vet visit, you could bet that 70 per cent of the people that would engage with the services would have a T [temporary] next to their name.’  ‘the demand for service is out there’  ‘[They had a] a campaign a few weeks, months ago maybe now where they had a vet here and they were doing vaccinations and de-sexing for free. I think a lot of people really liked that but the advertising of it, I don't think was very high’  ‘Even regular services, so say the vet comes on the first of every month or the next following day, even just have that regularly’  ‘On communication: ‘Yeah, and just letting everyone know when the vet's in town and what the vet's offering.’  ‘Ever since they’ve started this regular vet service, that comes up all the time, that’s made a massive difference, it really has’  ‘[We need] an increase in vet services….unfortunately they don't come - they come in our - the dry season only and that is probably from April/May through to maybe October/November. There are maybe two services during that time.’  ‘Without a vet down there, it's impossible’  ‘Well the reality is people - it's people's decision whether they take advantage of it or not.’  ‘I think the vet service was fantastic, I think they were good people. I think they provided a wonderful service’  ‘We had a - this was when I came here personally in 94, there was a vet here… She tried to get the [de-wormer] injected in dog whiskers so the dogs get healthier. The problem was then the dogs got healthier, then they'd have more litters. Then the problem just went around and around; so they stopped that. What they needed to do was probably have the dogs de-sexed. That probably would have worked out.’  ‘I know the de-sexing is free, but the other medication and that, they charge so people then tend to back away, because I don't know. I don't think they charge what you charge in the city, but they still charge.’  ‘Well, they read the papers, they listen to the news. Some of these are just word by mouth and by the time they get the news it's too late.’  On whether people are nervous of the vet: ‘No. Some of them got no transport to where the vets come.’  ‘We try to keep this locally, local people have the first priority of getting the animals de-sexed here.’  ‘Majority of time, when vet come up here, these contractors and teachers…..… they just came from down there, you should have get your dog de-sexed down there. Don't come up here and try to get the free service…. This afternoon, the locals come. They're busy. That vet is busy with them, their dog, so it always take up more time then’  ‘We want to make everything fair. We don't want to just do Bamaga, you know? We don't want other community to miss out. We want to do every community.’ |
| **Local dog management** | ‘Should have a register for all their pets. Collar and tag.’  ‘Council should get together and restrict number of dogs.’  ‘When the 2 dogs rule came in [in the 90s], they enforced that. There was hardly any dogs around. No stray dogs.’ | ‘A microchipping program or something would actually help minimise……those sorts of things. Then you're not actually intervening with people's dogs but at least you could track what's happening.’  ‘The Queensland Housing Policy, it states in the tenancy agreement that if you're not a hunter within the community, then you're only allowed two dogs per house. If you're a hunter, you're allowed five dogs. All has to be registered, right.’  ‘Well, council's going to put policy of two dogs per yard. That's not happening. Not being policed, I should say…… They just blame the environmental health.’  ‘there probably needs to be more education on getting the dogs, or maybe bylaws to say, all female dogs up here have got to be de-sexed.’  ‘So it's the two things of the population control, removing unwanted animals from the equation through the impounding process, getting people in - to control and better manage their animals themselves and then reducing that uncontrolled breeding in the population.’  ‘I think it’s all good vets coming up and all that, but all the animals - well, it's about animal control. I think the horses is the main one. I think taking the dogs as a problem, but a lot of people don't talk about the cats, which if you sit around late at night there's heaps there.’  ‘…trying to enforce the bylaws, which we had during pre-amalgamation … with amalgamation something died.’  ‘On dog management prior to council amalgamation: A major difference. I think the bylaws - well, council back then was strict on by the bylaws. So it was two dogs per house. It was really two dogs per house. The regular vets, they did de-sex them. But then the hunting ones had to be locked up properly and registered as well. So you might have six dogs.’  ‘community took pride back then being pre-amalgamation’  ‘I think we've got to start charging registration as well.’  ‘I think… the amalgamation didn't help us at all. We're very proud communities and everybody looks after one another. If there's a stray dog it will either be put down or taken back to his owner and all that. So trying to get that back into community is the hardest thing.’  ‘Yeah, well I want to know, what's the purpose of bringing the two-dog rule in. Well, is there an overcrowding of dogs in houses? Or in yards? I don't think there is. I'd really like to see the data on that if that's the case.’  ‘Some of them dogs got 12 dogs and that person don't go hunt. So, you can keep three dogs and get rid of them other dogs. Only if [who the] who's hunt, you can keep six dogs, seven dogs, but [impound], backyard, and yeah.’  ‘It's ridiculous, somebody got a truckload of dogs. What for? If you want that number of dogs then you need to keep them secure 24 hours unless you take them out somewhere. But yeah, they should enforce the law.’  ‘Do what every other council does, everyone is given a free house with a fence that works with gates, you can have two dogs, that's your limit. If they are caught outside and they're not registered, they go to the pound, if they're not picked up they're destroyed. Fixed, problem solved.’ |
